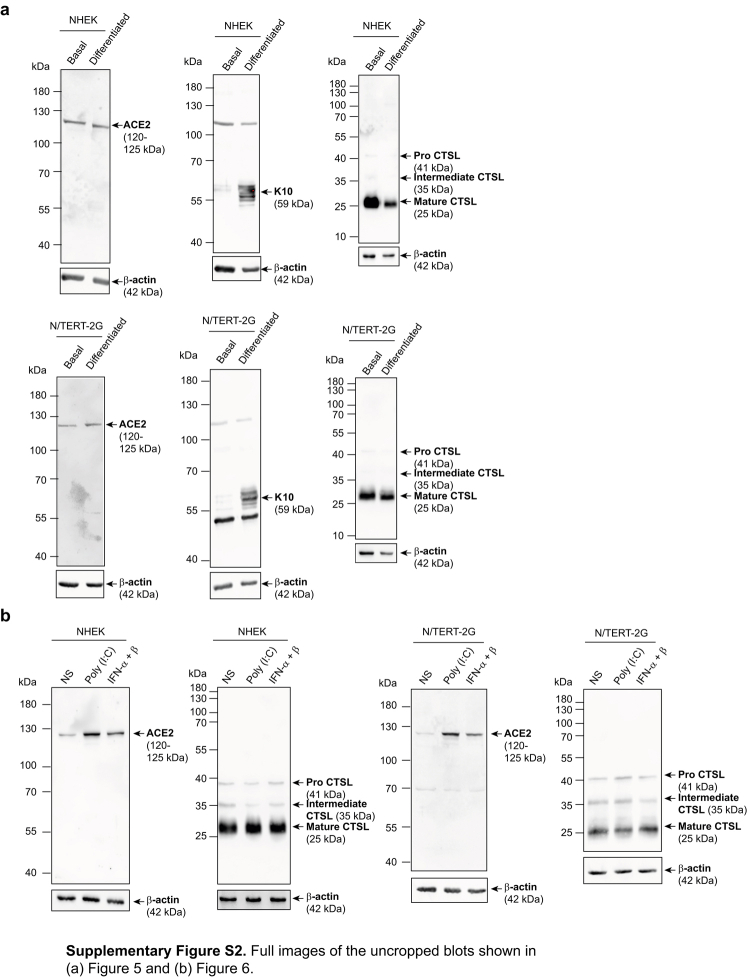# Human keratinocytes exhibit limited potential for SARS-CoV-2 infection despite ACE2 and mature cathepsin L expression

**DOI:** 10.1016/j.xjidi.2025.100447

**Published:** 2025-12-29

**Authors:** Leslie Hertereau, Manon Barthe, Noura Lamghari, Peggy Merida, Gaelle Pommier, Elisabeth Pinel, Jitendriya Swain, Delphine Muriaux, Hanan Osman-Ponchet, Véronique M. Braud

**Affiliations:** 1 Université Côte d’Azur, Centre National de la Recherche Scientifique (CNRS) UMR7275, Institut National de la Santé et de la Recherche médicale (INSERM) U1323, Institut de Pharmacologie Moléculaire et Cellulaire (IPMC), Valbonne, France; 2 PKDERM Laboratories, Valbonne, France; 3 IRIM UMR9004 CNRS, Université de Montpellier, Montpellier, France; 4 CEMIPAI UAR3725 CNRS, Université de Montpellier, Montpellier, France; 5 Department of Chemistry, School of Applied Sciences and Humanities, Vignan's Foundation for Science, Technology and Research, Guntur, India

**Keywords:** ACE2, Keratinocytes, SARS-CoV-2, Skin, Skin inflammation

## Abstract

The distribution of receptors and cellular factors across tissues determines differential susceptibility of cells to viral infection. For severe acute respiratory syndrome coronavirus 2, viral spike and nucleocapsid proteins have been detected in the skin of infected patients. Whether the virus can directly infect skin cells has yet to be fully evaluated. Severe acute respiratory syndrome coronavirus 2 enters cells through 2 routes: ACE2-driven endocytosis and TMPRSS2-mediated plasma membrane fusion or ACE2/alternative receptors-driven endocytosis and cathepsin L–dependent fusion. This study assessed the gene and protein expression of these entry receptors and coreceptors in primary keratinocytes and fibroblasts. We found that the main severe acute respiratory syndrome coronavirus 2 receptor ACE2 is present in human keratinocytes and is upregulated during their differentiation and toll-like receptor 3–mediated activation, whereas the coreceptor TMPRSS2 for fusion is absent, but mature cathepsin L is expressed. In vitro infection assays using the severe acute respiratory syndrome coronavirus 2 Delta variant showed that the virus can bind to the cell surface but cannot replicate within the cells. These findings suggest that although active viral replication in keratinocytes is unlikely, the presence and inducible upregulation of ACE2 in response to inflammatory stimuli may confer a limited potential for cutaneous viral entry, warranting further investigation into the consequences in terms of local inflammation and viral transmission.

## Introduction

As a barrier organ, the skin is continually challenged by environmental stressors that can lead to a wide range of disorders. Beyond sun exposure, factors such as pollution, microbial agents, and excessive washing contribute to skin dryness, barrier disruption, inflammation, and cancer. To counter these challenges, the skin has developed a robust immune surveillance system. Keratinocytes in the epidermis play a key role by sensing danger and initiating immune responses.

The COVID-19 pandemic raised the question of whether the skin might serve as an alternative entry route for severe acute respiratory syndrome coronavirus 2 (SARS-CoV-2), in addition to the primary transmission through the airways (Barthe et al, 2023; Xue et al, 2021). Indeed, the skin is inevitably exposed to the virus. Studies have shown that SARS-CoV-2 can be transmitted to human skin from wet or dry surfaces (Behzadinasab et al, 2021) and remain viable on human skin for up to 22 hours (Hirose et al, 2022). The implementation of strict hygiene measures has further impacted skin integrity, increasing the incidence of lesions and inflammation, potentially increasing the risk of percutaneous viral transmission (Hu et al, 2020; Mushtaq et al, 2021). Skin manifestations have been reported in SARS-CoV-2–infected individuals and were found predictive of infection (Brahimi et al, 2025; Visconti et al, 2021; Zhao et al, 2020). SARS-CoV-2 viral particles, spike, and nucleocapsid proteins have been detected in the skin of infected patients, although inconsistently (Barthe et al, 2023; Cazzato et al, 2022; Colmenero et al, 2020; Liu et al, 2020; Ma et al, 2022; Stein et al, 2022; Sun et al, 2020). Notably, spike protein depositions have been observed in autophagosomes within the skin capillary endothelia and found associated with SARS-CoV-2–induced vasculitic skin lesions (Gawaz et al, 2024). However, it remains uncertain whether such skin symptoms result from direct percutaneous infection or are secondary to systemic effects of the virus, such as vascular dissemination and induction of inflammation.

In this context, it is relevant to investigate further the mechanisms through which SARS-CoV-2 may enter the skin and whether local replication could occur.

Similar to many viruses, SARS-CoV-2 exhibits multiorgan tropism driven by the distribution of host cell receptors across different tissues (Baggen et al, 2021; Jackson et al, 2022). Although the respiratory tract is the primary target, the virus has been detected in other organs, including the skin (Barthe et al, 2023; Gupta et al, 2020). ACE2 has been identified as the primary entry receptor (Jackson et al, 2022; Walls et al, 2020; Yan et al, 2020). Viral entry by fusion involves the protease TMPRSS2, furin, and/or the transmembrane glycoprotein NRP1, which facilitate the entry of the virus through the host cell plasma membrane (Hoffmann et al, 2020; Shang et al, 2020). Alternatively, entry of the virus by endocytosis is mediated by ACE2 but can also be mediated by other alternative receptors, including CD147, DPP4/CD26, AXL, ASGR1, KREMEN1, and TMEM106B, and depends on the cathepsin L (CTSL) for endosomal membrane fusion (Baggen et al, 2023, 2021; Barthe et al, 2023; Bauer et al, 2012; Cheng et al, 2009; Ou et al, 2020; Peng et al, 2017; Shang et al, 2020; Zeeuwen et al, 2010). This diversity of viral entry pathways likely contributes to SARS-CoV-2’s broad tissue tropism and may be associated with long COVID-19 symptoms (Davis et al, 2023; Kaplan et al, 2021). Notably, infection of ACE2-negative cells has been observed, particularly in association with the E484D substitution in the spike protein (Baggen et al, 2023; Hoffmann et al, 2022; Niemeyer et al, 2022; Puray-Chavez et al, 2021; Yan et al, 2024). Moreover, pathological conditions can modulate the expression of the entry receptors, potentially enhancing susceptibility to infection (Chua et al, 2020; Krueger et al, 2021; Lin and Hong, 2022; Zhuang et al, 2020; Ziegler et al, 2020). ACE2 is known to be an IFN-stimulated gene, upregulated by IFN-α and IFN-γ (Smith et al, 2020; Ziegler et al, 2020). However, the functional relevance of this regulation is debated because a truncated isoform of ACE2 (designated as dACE2), which lacks spike binding affinity, is strongly upregulated by IFNs (Blume et al, 2021; Ng et al, 2020; Onabajo et al, 2020).

To date, a detailed expression profile of SARS-CoV-2 entry receptors and coreceptors in the skin has not been conducted. Such data are crucial to evaluate the risk of percutaneous infection. We therefore performed a comprehensive analysis of their expression in keratinocytes, fibroblasts, and skin explants under homeostatic and inflammatory conditions and correlated their expression with the susceptibility of keratinocytes to SARS-CoV-2 infection.

## Results

### Screen for SARS-CoV-2 entry receptors transcribed in healthy skin

**Figure 1:**
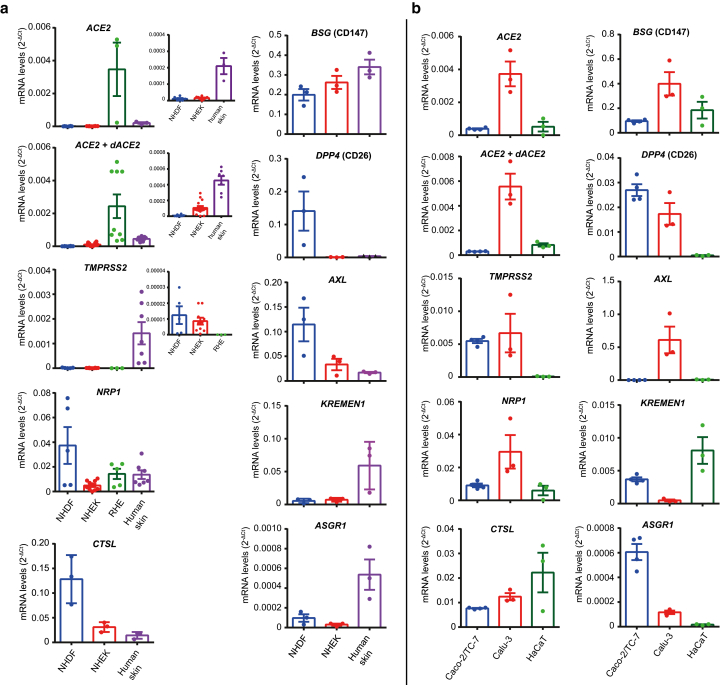
**Quantification of *ACE2*, *dACE2*, *TMPRSS2, NRP1, CTSL*, *BSG* (CD147), *DPP4* (CD26), *AXL*, *KREMEN1*, and *ASGR1* gene expression.** mRNA levels normalized to GAPDH (2^-ΔCT^) in (**a**) NHDF, NHEKs, RHE, and human skin samples and in (**b**) Caco-2/TC-7, Calu-3, and HaCaT cell lines. Gene expression was quantified by RT-qPCR in triplicate. Data represent the mean ± SEM from (**a**) 3 to 14 independent experiments using biological samples from different individuals and (**b**) 3 or 4 independent experiments from cultured cells. NHDF, normal human dermal fibroblast; NHEK, normal human epidermal keratinocyte; RHE, reconstructed human epidermis.

We first screened for the expression of transcripts encoding receptors and coreceptors implicated in SARS-CoV-2 entry, using various skin models: 2-dimensional primary normal human epidermal keratinocytes (NHEKs), 2-dimensional normal human dermal fibroblasts (NHDFs), 3-dimensional reconstructed human epidermis (RHE), and human skin explants (Figure 1a). We focused on *ACE2*, *TMPRSS2*, and *NRP1* involved in virus fusion to the plasma membrane and *CTSL*, *BSG* (CD147), *DPP4* (CD26), *AXL*, *ASGR1*, and *KREMEN1* implicated in endocytic uptake (Barthe et al, 2023). RT-qPCR assays were validated using Calu-3 and Caco-2/TC-7 cell lines, both known to express the receptors and compared with a human keratinocyte cell line, HaCaT (Figure 1b). *ACE2* mRNA levels were measured using 2 probes, one specific to the full-length *ACE2* transcript variant and another detecting both full-length and the truncated, IFN-inducible variant *dACE2.* Overall, very low levels of *ACE2* mRNA were measured in skin cells and explants. Specifically, *ACE2* and *dACE2* transcripts were undetectable in fibroblasts (NHDFs); present at low levels in undifferentiated keratinocytes (NHEKs); and more abundant in RHE, which contains differentiated keratinocytes (Figure 1a). This result raises the question of whether ACE2 expression is modulated during differentiation and/or in 3-dimensional cultures, as observed in airway epithelial cells, where ACE2 expression is elevated in differentiated cells (Jia et al, 2005). *TMPRSS2* was not detected in NHDF, NHEK, and RHE and was only weakly expressed in human skin explants (Figure 1a). This absence of expression was consistent with HaCaT cells, whereas *TMPRSS2* was readily detected in Caco-2/TC-7 and Calu-3 cells (Figure 1b). In contrast, *NRP1* transcripts were abundantly expressed in all 2-dimensional and 3-dimensional skin models as well as in the tested cell lines. Among the endocytic entry factors, *CTSL* (encoding CTSL) was significantly expressed in primary fibroblasts, keratinocytes, and skin explants. Similarly, high expression levels were observed for *BSG* (encoding CD147) and *AXL*, much lower levels were observed for *KREMEN1* and *DPP4*, and *ASGR1* mRNA was undetectable (Figure 1a). These results were generally consistent with expression patterns in HaCaT cells, except for *AXL*, which was expressed at lower level in HaCaT than in primary skin cells (Figure 1b).

In conclusion, our gene expression screen of SARS-CoV-2 entry receptors in human skin cells reveals that *ACE2* mRNA levels are low, with the highest expression observed in keratinocytes within RHE. *TMPRSS2* is largely undetectable, whereas *NRP1*, *CTSL*, *BSG* (CD147), *AXL*, and *DPP4* (CD26) are relatively abundant. This expression pattern suggests that SARS-CoV-2 may exploit ACE2 or alternative receptors such as CD147, AXL, and DPP4 as attachment receptors and a CTSL-mediated fusion in endosomes or internal membranes as entry mechanism.

### Expression of SARS-CoV-2 entry receptors and spike binding to primary keratinocytes support a possible endocytic viral entry mechanism

**Figure 2:**
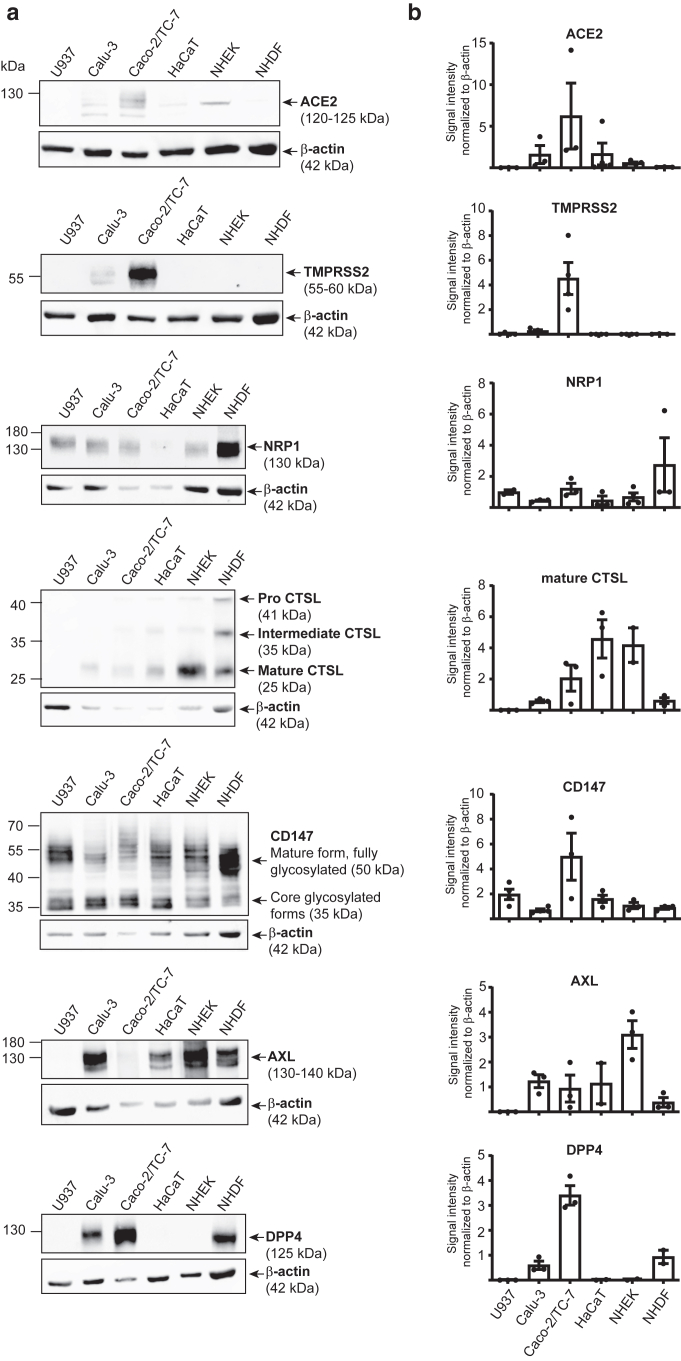
**Differential ACE2, TMPRSS2, NRP1, CTSL, CD147, AXL, and DPP4 protein expression.** Whole-cell lysates of the indicated cells were analyzed by western blot using the following antibodies: ACE2 (mAb clone AC384), TMPRSS2 (mAb clone S20014A), NRP1 (mAb clone 14H4), CTSL (mAb clone 33/1), CD147 (mAb clone HIM6), AXL (polyclonal goat IgG, AF154), and DPP4 (polyclonal goat IgG, AF1180). (**a**) Representative immunoblots from 2 to 4 independent experiments. (**b**) Densitometric quantification normalized to actin, expressed as mean ± SEM from 2 to 4 independent experiments. CTSL, cathepsin L.

**Supplementary Figure 1:**
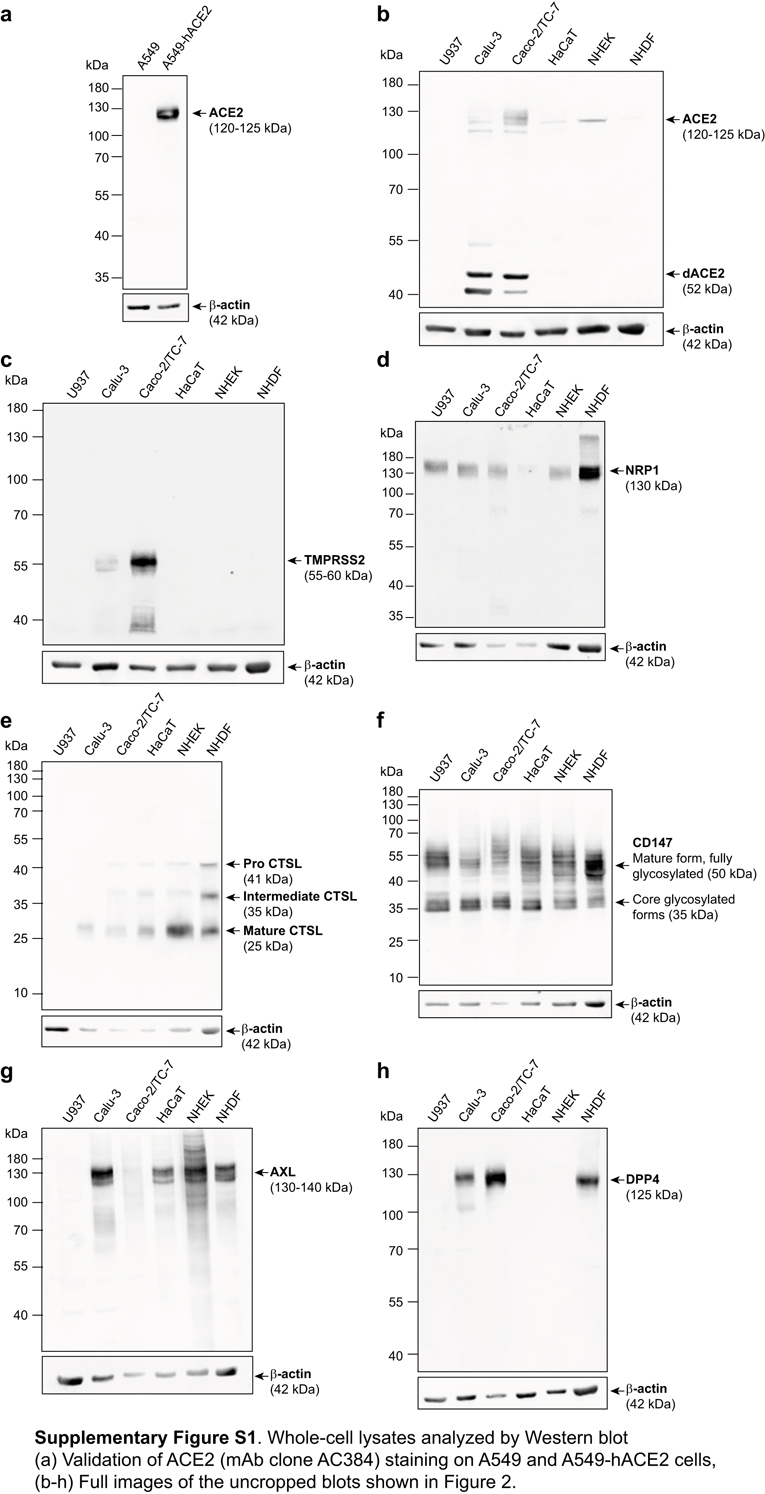


To validate the expression of SARS-CoV-2 entry receptors in the skin, we combined the quantification of their transcripts with the monitoring of their protein expression. Western blot analysis was performed using an antibody capable of detecting both the full-length glycosylated ACE2 isoform (120–125 kDa) and the short nonglycosylated dACE2 isoform (52 kDa) (Blume et al, 2021; Ng et al, 2020; Onabajo et al, 2020). Antibody specificity was validated using the human alveolar pulmonary A549 cell line transfected with human ACE2 (Laplantine et al, 2022; Swain et al, 2023) (Supplementary Figure S1a). As shown in Figure 2 and Supplementary Figure S1, both ACE2 isoforms were detected in the positive control cell lines Calu-3 and Caco-2/TC-7 but not in the negative control cell line U937. In line with transcript data, ACE2 protein levels were substantially lower in the HaCaT cell line and in primary keratinocytes (NHEKs). Interestingly, only the full-length ACE2 isoform, capable of binding to the viral spike, was detected in skin-derived cells (Supplementary Figure S1b). No ACE2 protein was detected in NHDFs, consistent with mRNA levels near the detection threshold (Figure 1a). TMPRSS2 protein was readily detected in Caco-2/TC-7 and Calu-3 but not in U937, HaCaT, NHEKs, or NHDFs, consistent with their extremely low *TMPRSS2* mRNA levels (Figure 2). In contrast, NRP1 was significantly expressed in all cell lines and primary skin cells, with much higher expression in fibroblasts than in keratinocytes and HaCaT, mirroring transcript data (Figure 2). CTSL was also detected in all cell types except U937, appearing in 3 forms: proenzyme, intermediate, and mature. In keratinocytes (NHEKs and HaCaT), the mature form predominated, indicating active enzymatic processing, whereas the pro and intermediate CTSL were predominant in NHDFs (Figure 2). CD147 was also highly expressed across all the cells tested. CD147 is heavily glycosylated, and both the mature form and the core glycosylated forms could be visualized, with variable levels of glycosylation between cells. AXL was detected in all cells except U937, with the highest expression observed in NHEKs. DPP4 was expressed in Calu-3, Caco-2/TC-7, and NHDFs but was absent in NHEKs, HaCaT, and U937 (Figure 2). Collectively, these findings confirm that all tested SARS-CoV-2 entry receptors, coreceptors, and proteases, except TMPRSS2, are expressed at the protein level in human skin.

**Figure 3:**
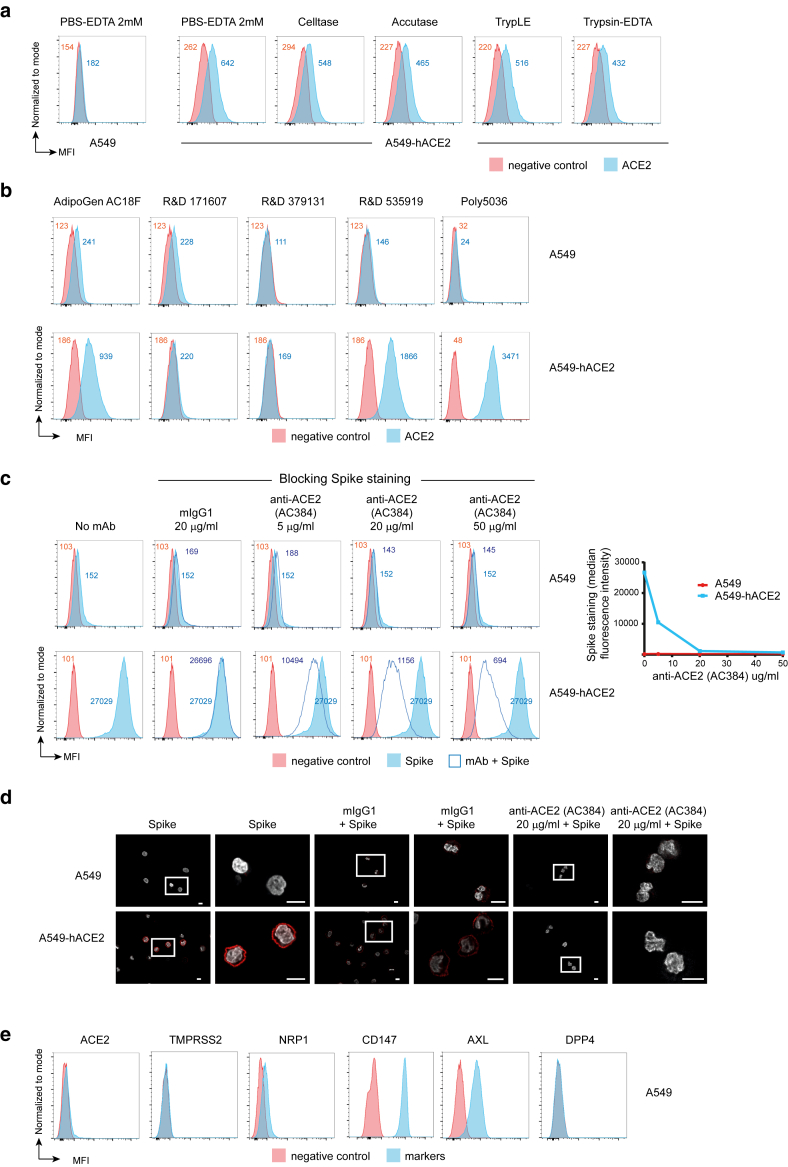
**Validation of flow cytometry protocols and anti-ACE2 antibodies.** (**a**) Comparison of cell detachment protocols. (**b**) Comparison of anti-ACE2 antibody staining by flow cytometry. (**c, d**) Binding of 2019-nCoV Spike Protein S1 (RBD) protein and blocking with anti-ACE2 antibody (mAb clone AC384) assessed by (**c**) flow cytometry and (**d**) immunofluorescence (red). Cell nuclei are stained with DAPI (white). Bar = 10 μm. Data are representative of 3 independent experiments. (**e**) Cell surface expression of ACE2 (mAb clone Poly5036), TMPRSS2 (mAb clone S20014A), NRP1 (mAb clone U21-1283), CD147 (mAb clone HIM6), AXL (mAb clone 108724), and DPP4 (mAb clone BA5b) in A549 cells. Data are representative of 2 independent experiments.

**Figure 4:**
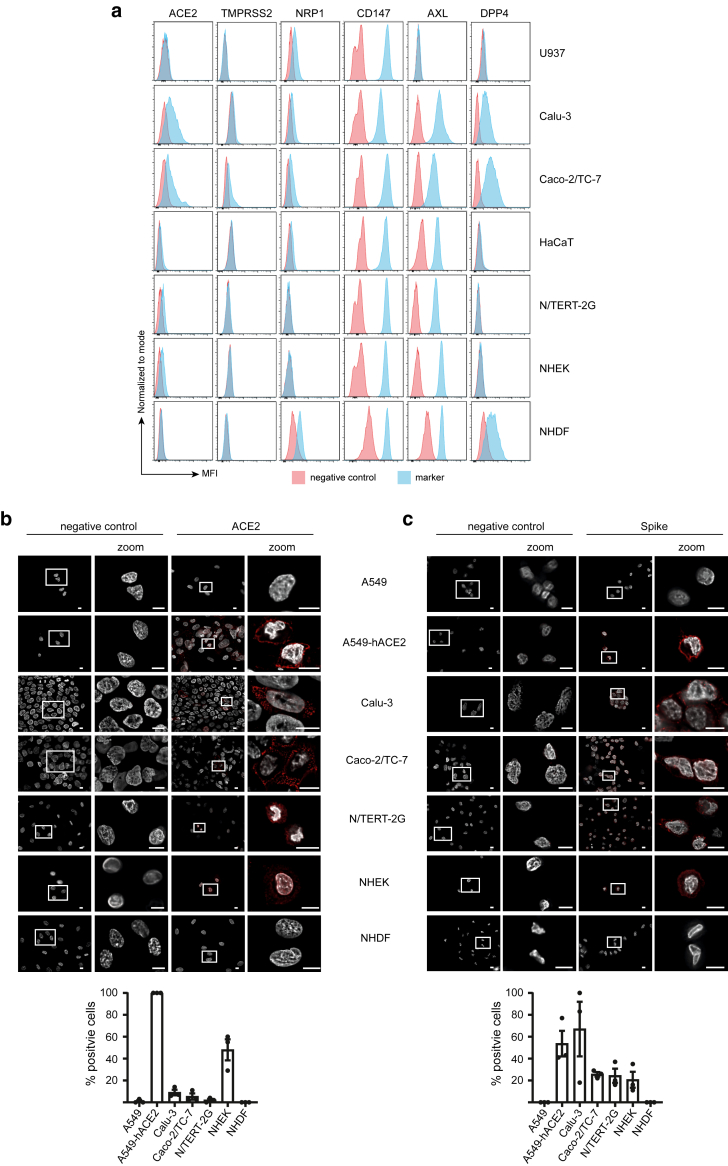
**Cell surface expression of ACE2, TMPRSS2, NRP1, CD147, AXL, and DPP4 receptors is associated with exclusive binding of SARS-CoV-2 Spike Protein S1 (RBD) to ACE2-expressing cells.** (**a**) Flow cytometry analysis of cell surface expression of ACE2 (mAb clone Poly5036), TMPRSS2 (mAb clone S20014A), NRP1 (mAb clone U21-1283), CD147 (mAb clone HIM6), AXL (mAb clone 108724), and DPP4 (mAb clone BA5b) in the indicated cell lines and primary skin cells. Data are representative of 2–4 independent experiments. (**b, c**) Representative immunofluorescence microscopy images showing (**b**) ACE2 expression (mAb clone Poly5036) and (**c**) binding of 2019-nCoV Spike Protein S1 (RBD) in red. Cell nuclei are stained with DAPI (white). Bar = 10 μm. The proportions of cells expressing ACE2 and binding to spike are shown. Data are representative of at least 2 independent experiments. SARS-CoV-2, severe acute respiratory syndrome coronavirus 2.

We next assessed whether they were expressed on the cell surface using flow cytometry, selecting cell detachment protocols that preserved the epitopes recognized by antibodies for each receptor. Although neither enzymatic nor nonenzymatic treatments affected antibody binding, nonenzymatic methods such as PBS-EDTA and citric saline buffer reduced keratinocyte viability and were thus excluded, leading to the use of Accutase throughout the study (Figure 3a). Flow cytometry staining closely matched protein detection of all the receptors by western blot (Figure 4a). Among the antihuman ACE2 mAbs reported to work in flow cytometry (Buchrieser et al, 2021; Sherman and Emmer, 2021), we identified 3 clones—AC18F (AdipoGen), 535919 (R&D Systems), and Poly5036 (BioLegend—that effectively stained A549-hACE2 cells but not the parental A549 cells (Figure 3b). However, only the Poly5036 antibody provided effective flow cytometry cell surface staining on primary cells (Figure 4a). Consistent with western blot analysis, the highest levels of ACE2 surface expression were detected in Calu-3 and Caco-2/TC-7 cells, with lower levels found in NHEKs and the immortalized N/TERT-2G, a keratinocyte cell line that shares more characteristics with NHEKs than with HaCaT cells (Figure 4a) (Smits et al, 2017). The Poly5036 antibody was also used to perform immunofluorescence (IF) staining, which confirmed ACE2 expression in A549-hACE2, Calu-3, Caco-2/TC-7, NHEK, and N/TERT-2G cells but not in parental A549 cells or NHDFs (Figure 4b). NHEKs were found to be sensitive to fixation performed prior to staining with the anti-ACE2 antibody, which caused diffusion of the signal within the cells. Binding of recombinant spike was therefore performed before fixation, providing a more specific staining pattern that mirrored ACE2 IF staining (Figure 4c). The specificity of spike binding was validated using a blocking anti-ACE2 mAb (clone AC384, Adipogen), which inhibited surface staining in a dose-dependent manner and also blocked IF staining (Figure 3c and d). Notably, spike failed to bind to NHDFs, which lack ACE2 but express high levels of CD147, AXL, and DPP4, receptors previously proposed as alternative SARS-CoV-2 entry receptors. Similarly, spike did not bind parental A549 cells, despite surface expression of CD147 and AXL (Figure 3e). These findings undermine the involvement of these alternative receptors in SARS-CoV-2 entry, an issue that has already been controversial (Baggen et al, 2021; Ragotte et al, 2021; Shilts et al, 2021).

In conclusion, the expression of ACE2, NRP-1, and CTSL, coupled with the absence of TMPRSS2, makes primary keratinocytes potentially permissive to SARS-CoV-2 infection through the endosomal route. In contrast, primary fibroblasts, which lack both ACE2 and TMPRSS2 and do not bind the spike protein, are unlikely to support viral entry or infection.

### Differentiation and toll-like receptor 3–mediated activation upregulate ACE2 expression in human epidermal keratinocytes

**Figure 5:**
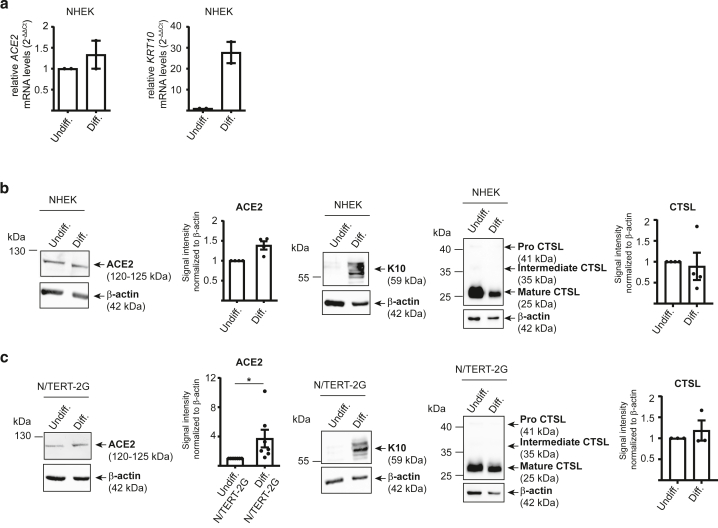
**Differentiation of human keratinocytes moderately upregulates ACE2.** NHEKs and N/TERT-2G cells were cultured as undifferentiated cells (denoted as undiff.) or differentiated cells (denoted as Diff.). (**a**) *ACE2* and *KRT10* mRNA levels relative to control (2^-ΔΔCT^), quantified by RT-qPCR in triplicate and normalized to GAPDH. Data represent the mean ± SEM from 2 independent experiments. Data were analyzed using the Wilcoxon signed-rank test to compare the 2 conditions. Only significant *P*-values (*P* < .05) are indicated in the figure. (**b, c**) ACE2, CTSL, and K10 protein expression analyzed by western blot using the following antibodies: ACE2 (mAb clone AC384), CTSL (mAb clone 33/1), and K10 (rabbit polyclonal Poly19054) in (**b**) NHEK and (**c**) N/TERT-2G cells. Shown are representative immunoblots and densitometric quantification, normalized to actin and expressed as mean ± SEM from 3 to 6 independent experiments. Data were analyzed using the Wilcoxon signed-rank test to compare the 2 conditions. Only significant *P*-values (*P* < .05) are indicated in the figure. ∗*P* < .05. CTSL, cathepsin L; K, keratin; NHEK, normal human epidermal keratinocyte.

The skin is constantly exposed to environmental stressors, mechanical injury, and microbial invasion, all of which can disrupt barrier integrity and initiate inflammatory responses. Epidermal lesions may also serve as potential entry points for viruses, including SARS-CoV-2. We therefore aimed to assess how ACE2 expression is modulated under physiological or inflammatory conditions. Elevated *ACE2* mRNA levels detected in RHE (Figure 1a) suggested that differentiated keratinocytes may express higher levels of ACE2. To investigate this, we cultured primary keratinocytes and the immortalized N/TERT-2G keratinocyte cell line in media with either low- or high-calcium concentrations to maintain an undifferentiated state or induce differentiation, respectively. Differentiation was validated by the induction of keratin 10. RT-qPCR analysis revealed a 1.5-fold increase in *ACE2* mRNA in calcium-differentiated NHEKs (Figure 5a), and western blot analysis showed a moderate but consistent increase in ACE2 protein expression in calcium-differentiated NHEKs and N/TERT-2G cells (Figure 5b and c and Supplementary Figure S2a). The level of expression of mature CTSL was not changed (Figure 5b and c). These results indicate that SARS-CoV-2 entry may be slightly enhanced in the upper epidermal layers, where keratinocytes are more differentiated.

**Figure 6:**
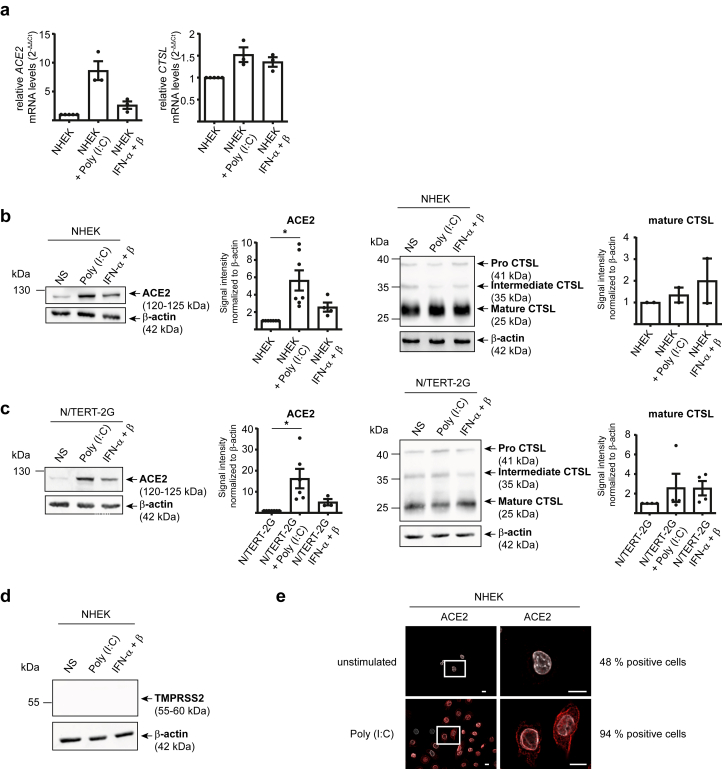
**TLR3-mediated activation upregulates ACE2 expression in human epidermal keratinocytes.** NHEK and N/TERT-2G cells were stimulated with Poly (I:C) and IFN-α + β for 24 hours. (**a**) ACE2 and CTSL mRNA levels relative to control (2^-ΔΔCT^), measured by RT-qPCR in triplicate and normalized to GAPDH. Data represent the mean ± SEM from 3 independent experiments. Data were analyzed using the Wilcoxon signed-rank test, comparing each stimulation condition with the unstimulated control. Only significant *P*-values (*P* < .05) are indicated in the figure. (**b, c**) Western blot analysis of ACE2 (mAb clone AC384) and CTSL (mAb clone 33/1). Shown are representative immunoblots and densitometric quantification, normalized to actin and expressed as mean ± SEM from 3 to 6 independent experiments. Data were analyzed using Wilcoxon signed-rank test, comparing each stimulation condition with the unstimulated control. Only significant *P*-values (*P* < .05) are indicated in the figure. ∗ *P* < .05. (**d**) Western blot analysis of TMPRSS2 (mAb clone S20014A) expression. Shown is immunoblot representative of 2 independent experiments. (**e**) Representative immunofluorescence microscopy images showing ACE2 expression (mAb clone Poly5036) (red). Cell nuclei are stained with DAPI (white). Bar = 10 μm. Data are representative of at least 2 independent experiments. CTSL, cathepsin L; NHEK, normal human epidermal keratinocyte; Poly (I:C), polyinosinic:polycytidylic acid; TLR3, toll-like receptor 3.

In addition to differentiation, inflammatory signaling can modulate keratinocyte gene expression. Skin inflammation is primarily driven by damage-associated molecular patterns released during tissue injury and pathogen-associated molecular patterns derived from microorganisms. UV light has been shown to activate toll-like receptor 3 (TLR3) in keratinocytes through the recognition of self-derived double-stranded RNAs (Bernard et al, 2012; Lai et al, 2009). On the basis of these findings, we investigated whether the synthetic double-stranded RNA analog polyinosinic:polycytidylic acid (Poly [I:C]), a potent TLR3 agonist, could also influence ACE2 expression. As shown in Figure 6a, Poly (I:C) treatment induced an 8-fold increase in *ACE2* mRNA expression in NHEK cells and ∼6 and ∼15-fold statistically significant increase in ACE2 protein expression in NHEK and N/TERT-2G cells, respectively (Figure 6b and c and Supplementary Figure S2b). By contrast, the expression of mature CTSL remained unchanged, and TMPRSS2 remained undetectable (Figure 6a–d). The upregulation of ACE2 in Poly (I:C)–stimulated NHEKs was also visualized by IF (Figure 6e). To investigate the mechanisms involved, we screened a panel of cytokines secreted by keratinocytes in response to double-stranded RNA stimulation and identified IFN-α and IFN-β as key contributors to ACE2 induction, consistent with previous reports (Smith et al, 2020; Ziegler et al, 2020). IFN-α and IFN-β stimulation was however less efficient than Poly (I:C) stimulation (Figure 6a–c).

Collectively, these findings highlight that skin inflammation, particularly through TLR3 activation, promotes ACE2 expression and spike binding in primary keratinocytes, potentially enhancing SARS-CoV-2 viral entry in keratinocytes.

### SARS-CoV-2 infection of human epidermal keratinocytes in vitro

**Figure 7:**
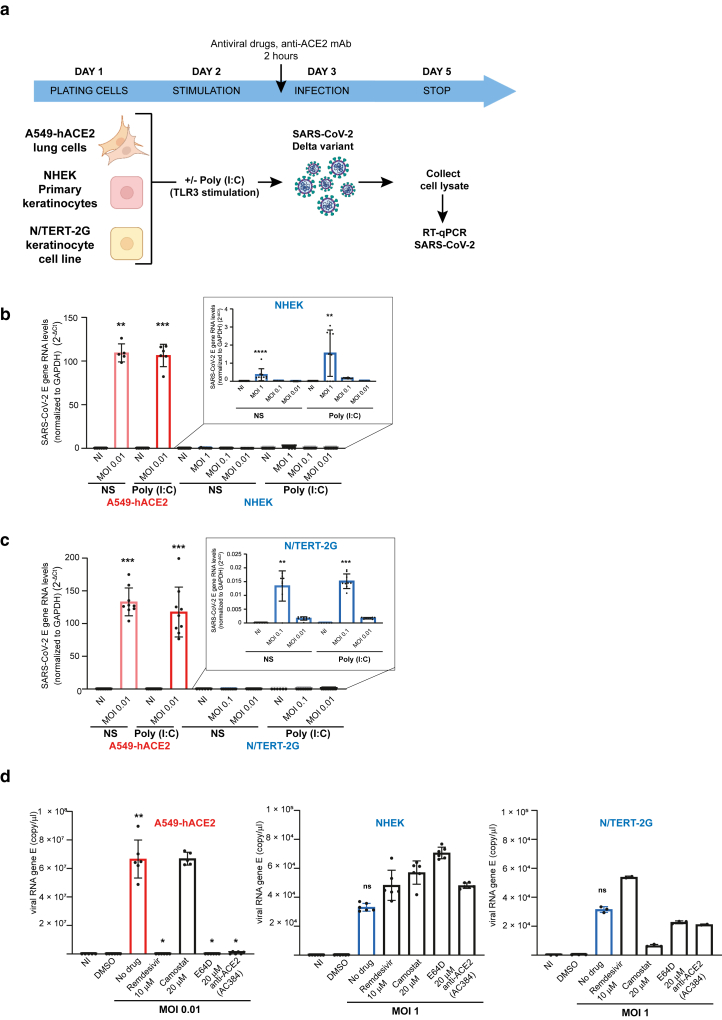
**SARS-CoV-2 Delta variant (B.1.617.2) binds to human epidermal keratinocytes but does not replicate.** (**a**) Experimental design of SARS-CoV-2 Delta variant (B.1.617.2) infection. (**b, c**) A549-hACE2, (**b**) NHEK, and (**c**) N/TERT-2G cells unstimulated or stimulated with Poly (I:C) were noninfected (denoted as NI) or infected for 48 hours at the indicated MOI with SARS-CoV-2 Delta variant (B.1.617.2). SARS-CoV-2 E gene RNA levels normalized to GAPDH (2^-ΔCT^) were calculated from triplicates of 1 or 2 replicates and expressed as mean values ± SD. Data were analyzed using the Kruskal–Wallis 1-way ANOVA test, comparing each infected condition with the noninfected control (denoted as NI). Only significant *P*-values (*P* < .05) are indicated above the infected conditions in the figure. ∗∗∗∗*P* < .0001, ∗∗∗*P* < .001, and ∗∗*P* < .01. (**d**) A549-hACE2, NHEK, and N/TERT-2G cells were noninfected (denoted as NI) or infected for 72 hours with SARS-CoV-2 Delta variant (B.1.617.2) in the absence or presence of antiviral drugs or blocking anti-ACE2 mAb (clone AC384) as indicated. SARS-CoV-2 RNA gene E (copy/μl) were calculated from triplicates of 1 or 2 replicates and expressed as mean values ± SD. Data were analyzed using the Kruskal–Wallis 1-way ANOVA test, comparing each infected condition without drug with the noninfected DMSO control and comparing each infected condition with drug with the corresponding infected condition without drug. Only significant *P*-values are indicated above the infected conditions in the figure. ∗∗*P* < .001 and ∗*P* < .05. MOI, multiplicity of infection; NHEK, normal human epidermal keratinocyte; Poly (I:C), polyinosinic:polycytidylic acid; SARS-CoV-2, severe acute respiratory syndrome coronavirus 2.

**Figure 8:**
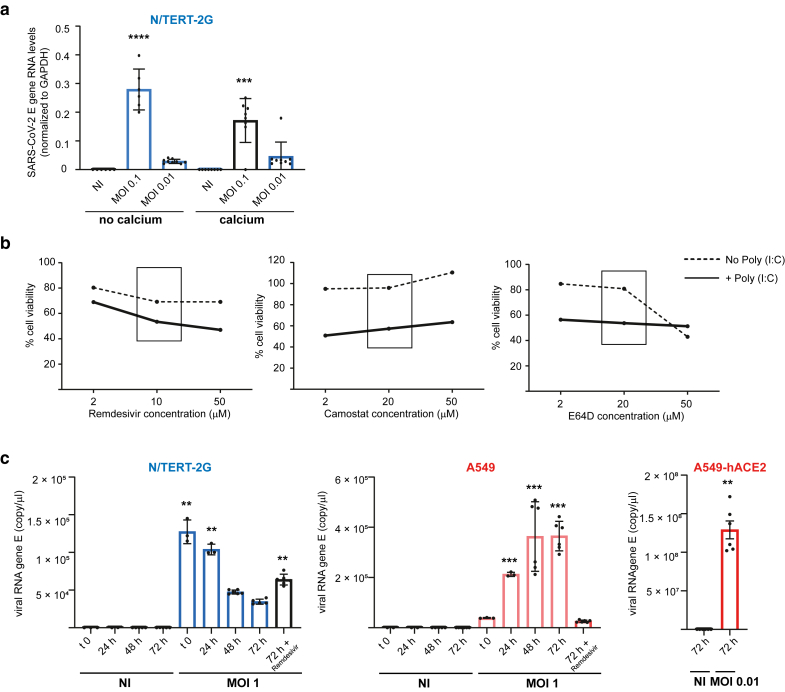
**SARS-CoV-2 Delta variant (B.1.617.2) infection of human epidermal keratinocytes in vitro.** (**a**) N/TERT-2G cells, either undifferentiated or calcium differentiated, were noninfected (denoted as NI) or infected for 48 hours at the indicated MOI with SARS-CoV-2 Delta variant (B.1.617.2). SARS-CoV-2 E gene RNA levels normalized to GAPDH (2^-ΔCT^) were calculated from triplicates of 1 or 2 replicates and expressed as mean values ± SD. Data were analyzed using the Kruskal–Wallis 1-way ANOVA test, comparing each infected condition with the noninfected (denoted as NI) control. Only significant *P*-values (*P* < .05) are indicated above the infected conditions in the figure. ∗∗∗∗*P* < .0001 and ∗∗∗*P* < .001. (**b**) Cell viability of N/TERT cells in the presence of increasing concentrations of antiviral drugs as indicated. (**c**) Kinetics of SARS-CoV-2 Delta variant (B.1.617.2) infection of N/TERT-2G and A549 cells at high MOI of 1, with A549-hACE2 as control at low MOI of 0.01, 72 hours pi. SARS-CoV-2 RNA gene E (copy/μl) were calculated from triplicates of 1 or 2 replicates and expressed as mean values ± SD. Data were analyzed using the Kruskal–Wallis 1-way ANOVA test, comparing each infected condition with their corresponding noninfected (denoted as NI) control. Only significant *P*-values (*P* < .05) are indicated above the infected conditions in the figure. ∗∗∗*P* < .001 and ∗∗*P* < .01. MOI, multiplicity of infection; pi, postinfection; SARS-CoV-2, severe acute respiratory syndrome coronavirus 2.

**Figure 9:**
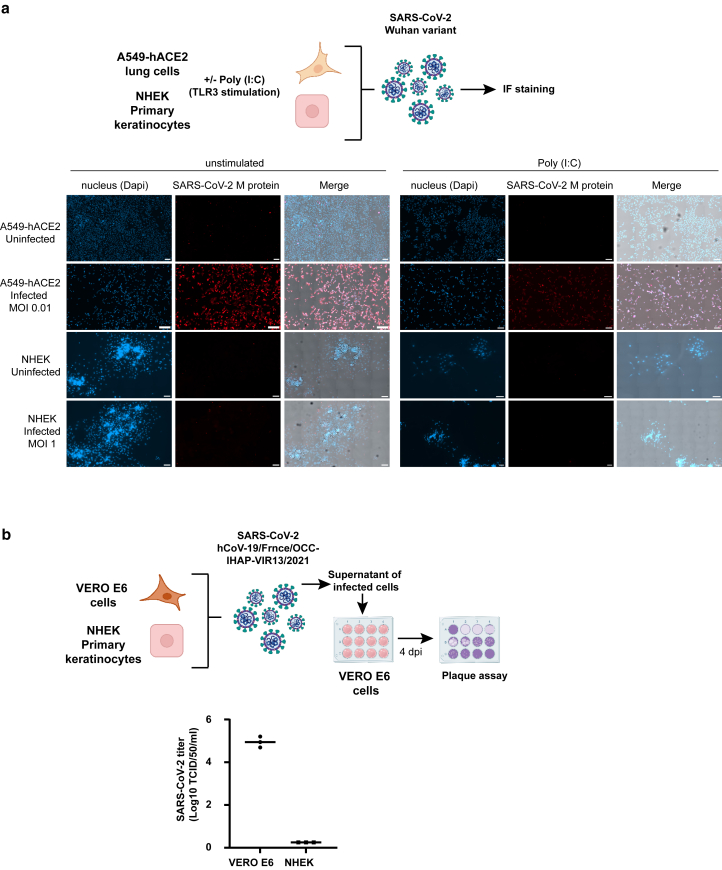
**SARS-CoV-2 Wuhan and hCoV-19/France/OCC-IHAP-VIR13/2021 variant infection of human epidermal keratinocytes in vitro.** (**a**) Confocal images showing noninfected and SARS-CoV-2 Wuhan variant–infected cells. A549-hACE2 and NHEK cells were stimulated with Poly (I:C) for 24 hours. A549-hACE2 cells were infected at MOI of 0.01, and NHEK cells were infected at MOI of 1 for 2 hours, washed, and incubated in culture medium for 72 hours. Cells were stained with anti–SARS-CoV-2 M protein and secondary AF568-conjugated secondary antibody (red) as described in Assou et al (2023). Nuclei were counterstained with DAPI (blue). Bar = 20 μm. (**b**) VERO E6 and NHEK cells were infected at MOI of 0.5 with SARS-CoV-2 strain hCoV-19/France/OCC-IHAP-VIR13/2021 for 2 hours, and the cells were washed 3 times and incubated in culture medium for 72 hours. Viral titers in the collected supernatants were determined by standard cytopathic effect assay on VERO E6 using Spearman & Käber algorithm and expressed as 50% Tissue Culture Infectious Dose (TCID50)/ml (NeoVirTech, Toulouse, France). MOI, multiplicity of infection; NHEK, normal human epidermal keratinocyte; Poly (I:C), polyinosinic:polycytidylic acid; SARS-CoV-2, severe acute respiratory syndrome coronavirus 2.

Given the expression of ACE2 and mature CTSL in keratinocytes and the upregulation of ACE2 after calcium-induced differentiation and TLR3 stimulation, we investigated whether NHEK and N/TERT-2G cells could be infected by SARS-CoV-2 in vitro (Figure 7a). We used the Delta variant (B.1.617.2), which has been associated with more frequent cutaneous manifestations during the Delta wave of the COVID-19 pandemic (Visconti et al, 2022). SARS-CoV-2 envelope (denoted as E) gene RNA levels were quantified 48 hours after infection in cell lysates and were significantly elevated compared with those of their respective uninfected controls. In infected A549-hACE2 control cells (at low multiplicity of infection [MOI] = 0.01), E gene RNA levels were ∼100-fold higher than those in NHEKs (at high MOI = 1) and in N/TERT-2G (MOI = 0.1–0.01) (Figure 7b and c). Stimulation with Poly (I:C) did not enhance infection in NHEK and N/TERT-2G cells, despite inducing ACE2 expression at the cell surface (Figure 6). Similarly, calcium-induced differentiation had no effect on viral susceptibility of N/TERT-2G cells (Figure 8a). To validate these findings, we performed functional blocking experiments using remdesivir (to inhibit viral replication), camostat mesylate (to block TMPRSS2-dependent entry), E64D (to block CTSL-dependent entry), and the anti-ACE2 mAb (clone AC384) to block spike–ACE2 binding. A cell viability test confirmed that the drug concentrations used for A549-hACE2 cells (Laplantine et al, 2022) were not significantly toxic for keratinocytes (Figure 8b). As shown in Figure 7d, all treatments except camostat mesylate significantly reduced infection in A549-hACE2 cells, consistent with the lack of TMPRSS2 expression observed in this cell line (Laplantine et al, 2022). This also confirms that the virus enters these cells by an ACE2-dependent endocytosis and further replicates. In NHEK and N/TERT-2G cells, viral RNA levels remained comparable with those of the input virus across all conditions, at high MOI, and in the presence of any of the inhibitors (Figure 7d), indicating that although the Delta strain can bind to the cell surface, it does not replicate in these cells. This lack of viral replication was confirmed by a time-course analysis of viral RNA levels 0, 24, 48, and 72 hours after infection, showing that the virus did not replicate in N/TERT-2G (MOI = 1), because RNA levels over time remained lower than the control at t0. Low levels of replication were detected over time in A549 cells (MOI = 1), likely owing to some viral uptake, whereas a low MOI of 0.01 was sufficient to achieve high viral load in permissive A549-hACE2 cells (Figure 8c). To further confirm that SARS-CoV-2 does not replicate in keratinocytes, we performed IF staining followed by confocal microscopy imaging, comparing infection in A549-hACE2 control cells at low MOI with infection in NHEK cells at high MOI, using a Wuhan strain. Cells were either unstimulated or stimulated with Poly (I:C) prior infection. The anti-M antibody failed to detect viral signal above background levels in noninfected A549-hACE2 as well as in both noninfected and infected NHEK cells, whereas clear M protein staining was visualized in the majority of infected A549-hACE2 cells (Figure 9a). Moreover, Poly (I:C) stimulation did not modulate the observed infection pattern of unstimulated cells. Similarly, no replication was observed using another SARS-CoV-2 strain, the SARS-CoV-2 hCoV-19/France/OCC-IHAP-VIR13/2021, which was not produced by in vitro infected NHEKs (Figure 9b).

## Discussion

SARS-CoV-2 primarily infects the respiratory tract, but viral RNA or particles have also been detected in extrapulmonary sites, including the skin (Barthe et al, 2023; Cazzato et al, 2022; Colmenero et al, 2020; Gupta et al, 2020; Liu et al, 2020; Ma et al, 2022; Stein et al, 2022; Sun et al, 2020). Cutaneous manifestations have been widely reported in COVID-19, particularly during the Delta wave (Visconti et al, 2022), and skin manifestations have also been proposed as predictors of infection with higher sensitivity than nasopharyngeal swab tests (Visconti et al, 2021). However, whether skin cells are permissive to productive SARS-CoV-2 infection remains an open question.

In this study, we provide evidence that primary human keratinocytes express ACE2 and mature CTSL but lack TMPRSS2 expression. This is in agreement with previous reports of ACE2 and CTSL expression in the skin (Cheng et al, 2009; Hamming et al, 2004; Kaplan et al, 2021; Sun et al, 2020; Xue et al, 2021; Zeeuwen et al, 2010) and low TMPRSS2 expression (Cazzato et al, 2022; Ma et al, 2020). These findings suggest that SARS-CoV-2 may enter skin cells through the endosomal pathway rather than through plasma membrane fusion. Although in vitro infection assays demonstrate that the virus can bind to and potentially enter primary keratinocytes, it fails to replicate, indicating a restriction at entry or shortly thereafter. Thus, efficient viral propagation in keratinocytes appears unlikely.

Given that ACE2, the primary entry receptor, is significantly expressed at the protein level in primary keratinocytes and that CTSL is present predominantly in its mature, active form, SARS-CoV-2 may be internalized through clathrin-mediated endocytosis and subsequent endosomal membrane fusion. This is consistent with the fact that endocytosis is a common mechanism of viral entry across epithelial barriers (Bomsel and Alfsen, 2003). Moreover, the ability of SARS-CoV-2 to survive on human skin for up to 22 hours (Behzadinasab et al, 2021; Hirose et al, 2022) provides a temporal window for viral contact and uptake. Interestingly, we did not detect TMPRSS2 expression in human skin, suggesting that the virus cannot use the fusion with the plasma membrane in the skin, unless other TMPRSS proteases participate in the process and compensate for the lack of TMPRSS2. Notably, TMPRSS13 is predominantly expressed in human skin and has been shown to enhance cellular viral uptake and replication of SARS-CoV-2 (Kishimoto et al, 2021; Stevaert et al, 2022). Its role in the skin may be further examined in the context of SARS-CoV-2 infection. Several alternative entry receptors have also been proposed (Baggen et al, 2023, 2021; Barthe et al, 2023). We detected high surface expression of CD147, AXL, and DPP4 receptors on keratinocytes. However, this was not sufficient to render cells permissive to infection. The involvement of these alternative receptors remains controversial. Consistent with previous findings (Ragotte et al, 2021; Shilts et al, 2021), we obtained no binding of spike to ACE2-negative but CD147, AXL- or DPP4-positive skin fibroblasts or control A549 cells, reinforcing the primary role of ACE2 in SARS-CoV-2 skin tropism. Nonetheless, whether additional receptors contribute to viral entry in the presence or absence of ACE2 requires further study. Notably, during the course of our study, 2 independent groups reported that TMEM106B acts as an alternative receptor mediating ACE2-independent SARS-CoV-2 infection in vitro (Baggen et al, 2023; Yan et al, 2024). Because we observed that SARS-CoV-2 failed to replicate in keratinocytes, we did not further investigate TMEM106B expression in our study. Interestingly, Ma et al (2022) established a skin organoid model using human pluripotent stem cells and visualized spike in infected organoids within hair follicle. They reported that SARS-CoV-2 primarily targeted keratin 17^+^ and, to a lesser extent, keratin 71^+^, PDGFRα^+^ hair follicles as well as a subset of neurons. Consistent with our findings, they demonstrated that the virus could bind to specific types of keratinocytes within the organoids. Their observation of hair follicle loss upon infection, associated with DNA damage, suggests that there may be some degree of viral replication within keratin 17^+^ keratinocytes, although this still needs to be clearly demonstrated.

The mechanisms underlying SARS-CoV-2 skin infection may vary under conditions of environmental or physiological stress. We found that ACE2 expression was upregulated in differentiated keratinocytes, similarly to airway epithelial cells (Jia et al, 2005), and upon TLR3 and type I IFN stimulation. *ACE2* has previously been reported to be a human IFN-stimulated gene (Smith et al, 2020; Ziegler et al, 2020), although much of the signal detected the induction of a truncated form of ACE2, that is dACE2, that does not bind to spike (Blume et al, 2021; Ng et al, 2020; Onabajo et al, 2020). In the skin, only the full-length ACE2 protein binding to spike is expressed and is induced by Poly (I:C). Stimulation of TLR3 in keratinocytes is central in the homeostasis of the skin, being part of the processes of inflammation in response to infection and injury. Not only double-stranded RNA viruses but also double-stranded RNA released from necrotic or dying keratinocytes that have been exposed to UV light can stimulate TLR3 (Bernard et al, 2012; Lai et al, 2009), thus upregulating simultaneously ACE2. Therefore, compromised and inflamed skin would be more permissive to SARS-CoV-2, in line with high ACE2 expression reported in skin lesions of patients with psoriasis (Tembhre et al, 2021; Xu et al, 2022). Viral penetration may be tested in ex vivo models of damaged human skin (Barthe et al, 2024), similarly to infection of iALI cultures (Assou et al, 2023). This raises a paradox because the drastic protective hygiene measures recommended by the World Health Organization to reduce fomite transmission may simultaneously damage the skin barrier, inadvertently enhancing its vulnerability to SARS-CoV-2 through the upregulation of ACE2 and disruption of epidermal integrity. Furthermore, the virus itself may increase ACE2 expression through TLR3 and type I IFN activation. However, this does not lead to productive infection, possibly because of concomitant induction of antiviral IFN responses (Samuel, 2023). Keratinocytes may express IFN-stimulated genes upon infection, resulting in a viral restriction that can target viral entry. Alternatively, keratinocytes may lack additional cofactors required for replication (Baggen et al, 2021). Further studies are needed to understand why keratinocytes are not permissive.

We infected keratinocytes in vitro with a SARS-CoV-2 Delta variant because of its high transmissibility, higher intrinsic severity (Twohig et al, 2022), and its association with increased dermatologic manifestations (Visconti et al, 2022). Although the virus was able to bind to keratinocytes, no productive replication was observed. This finding suggests that cutaneous symptoms may arise from local inflammatory responses rather than direct cytopathic effects due to viral replication. Endothelial injury induced by SARS-CoV-2 has also been proposed as a contributing mechanism (Brahimi et al, 2025). For comparison, we tested early SARS-CoV-2 strains, which failed to replicate in keratinocytes, thereby attenuating the importance of variant-specific differences in cellular tropism. In addition, a previous study evaluated infection of NHEKs with an Omicron variant, which similarly did not replicate; however, those experiments lacked a positive control of infection (Zupin et al, 2023).

In conclusion, our findings demonstrate that primary human keratinocytes, despite expressing ACE2 and mature CTSL and exhibiting robust SARS-CoV-2 spike binding, do not support detectable productive viral replication, highlighting their limited susceptibility to infection. Furthermore, inflammation-induced upregulation of ACE2 (eg, through Poly [I:C]) enhances viral interaction with keratinocytes. We therefore propose that SARS-CoV-2 may exploit compromised skin barriers, including macroscopic wounds (eg, lacerations) or microscopic defects (eg, inflammation-induced microtears, UV-induced damage), or hair follicles (Ma et al, 2022) to access the epidermis through endocytosis- or transcytosis-mediated mechanisms. Similar pathways have been described in other epithelial tissues, such as the intestinal epithelium (Clerbaux et al, 2022) and the brain (Pepe et al, 2022; Song et al, 2021). Although keratinocytes do not support viral replication, they may act as transient reservoirs that facilitate viral dissemination, similarly to the tunneling nanotubule-mediated spread of SARS-CoV-2 from permissive to nonpermissive cells observed in the brain (Pepe et al, 2022). Importantly, as SARS-CoV-2 continues to evolve, sustained genomic and clinical surveillance is essential to detect emerging variants with increased pathogenicity or enhanced skin tropism.

## Materials and Methods

### Cell lines and primary skin samples

Human lung adenocarcinoma Calu-3 (Sterlab, Vallauris, France), human colon adenocarcinoma Caco-2/TC-7 (Sigma-Aldrich, St-Quentin-Fallavier, France), human epidermal keratinocyte cell line HaCaT (CLS, Köhln, Germany), and human promonocytic myeloid leukemia cell line U937 (ATCC, Manassas, VA) were maintained in complete MEM, IMDM, or DMEM medium (Gibco-Thermo Fisher Scientific, Courtaboeuf, France) supplemented with 10% fetal calf serum (Pierce, Thermo Fisher Scientific), penicillin (100 IU/ml), and streptomycin (100 μg/ml) (Gibco-Thermo Fisher Scientific) at 37 °C in a humidified atmosphere with 5% carbon dioxide. Human pulmonary alveolar A549 (ECACC, Salisbury, United Kingdom) and A549-hACE2 cells were previously described and maintained in complete RPMI (Laplantine et al, 2022; Swain et al, 2023). The human N/TERT-2G keratinocyte cell line was obtained from Yves Poumay Laboratory (Research Unit of Molecular Physiology, Namur Research Institute for Life Sciences), courtesy of the J. Rheinwald laboratory (Harvard Medical School) (Dickson et al, 2000). N/TERT-2G cells were cultured in CnT-PR medium (CellnTec Advanced Cell Systems, Bern, Switzerland) and maintained below confluence to promote proliferation and prevent differentiation. Upon reaching confluence, the medium was replaced with CnT-PR-D medium supplemented with 1.2 mM calcium chloride solution (Sigma-Aldrich), and the cells were cultured for an additional 5–6 days to induce differentiation.

Human skin samples, NHEKs, RHE, and NHDFs were purchased from Sterlab. Skin samples were obtained from abdominal plastic resection surgery, with the patient’s informed consent, in accordance with the Declaration of Helsinki (Sterlab AC-2023-5620), and under French Ethics Committee approvals (DC-2023-5457 and DC-2018-3312). Human primary keratinocytes (passages 1–3) were maintained in keratinocyte culture medium (Sterlab) below confluence to prevent differentiation. Upon confluence, differentiation was induced using the same medium supplemented with 1.2 mM calcium chloride solution (Sigma-Aldrich), and cells were analyzed after 5–6 days of culture. Human primary fibroblasts (passages 1–9) were cultured in DMEM supplemented with 10% fetal calf serum, penicillin (100 IU/ml), and streptomycin (100 μg/ml).

### In vitro Poly (I:C) and cytokine stimulations

The N/TERT-2G keratinocyte cell line and NHEK cells were stimulated for 24 hours in medium supplemented with Poly (I:C) at 10 μg/ml (Invivogen, Toulouse, France), IFN-α, and IFN-β alone or in combination at 10 ng/ml (Peprotech, Neuilly-sur Seine, France).

### RNA isolation and RT-qPCR

#### RNA extraction

Total RNA was isolated using the SV Total RNA isolation system (Promega, Lyon, France) according to the manufacturer’s instructions. For skin samples, biopsies were cut into small pieces, mixed with D lysis matrix (MP Biomedicals) in RNA lysis buffer, and homogenized for 3 cycles of 30 seconds at 6 m/s using the FastPrep-24 5G homogenizer (MP Biomedicals). RNA concentrations were quantified using a NanoDrop ND-1000 spectrophotometer (NanoDrop, Thermo Fisher Scientific) or a BioPhotometer plus (Eppendorf, Montesson, France).

#### Reverse transcription

A total of 500 ng of total RNA was reverse transcribed into cDNA using the High-Capacity RNA-to-cDNA Master Mix kit (Applied Biosystems, Thermo Fisher Scientific), following the manufacturer’s instructions.

#### qPCR

PCR was performed using TaqMan Universal PCR Master Mix II, no UNG (Applied Biosystems, Thermo Fisher Scientific) on a LightCyclerv480/1536 (Roche, Meylan, France) or a 7500 Real-Time PCR System (Applied Biosystems, Thermo Fisher Scientific). Polymerase activation was carried out for 10 minutes at 95 °C, followed by 40 cycles of denaturation (15 seconds at 95 °C) and annealing/extension (1 minute at 60 °C). The following TaqMan MGB-FAM-labelled probes (Applied Biosystems, Thermo Fisher Scientific) were used for qPCR analysis: *ACE2* (Hs01085340_m1), *ACE2 + dACE2* (Hs01085333_m1), *TMPRSS2* (Hs01120965_m1), *NRP1* (Hs00826128_m1), *CTSL* (Hs00964650_m1), *BSG* (CD147) (Hs00936295_m1), *DPP4* (Hs00897386_m1), *AXL* (Hs01064444_m1), *KREMEN1* (Hs00230750_m1), *ASGR1* (Hs01005019_m1), and reference gene *GAPDH* (Hs02758991_g1). Gene expression was normalized to GAPDH, and mRNA expression levels were calculated using the comparative cycle threshold (Ct) method (2^−ΔCt^). Target and reference genes were amplified in separate reactions. Each PCR reaction was performed in triplicate in a final volume of 25 μl. PCR fluorescence data were analyzed using LightCycler 480 Software (version 1.5) (Roche) or 7500 software (version 2.3) (Applied Biosystems).

### Western blotting

Whole-cell extracts were prepared in RIPA lysis buffer (50 mM Tris-hydrogen chloride, pH 8, 0.5% sodium deoxycholate, 2 mM EDTA, 1% IGEPAL CA-630, 150 mM sodium chloride, 0.1% SDS), supplemented with 5 mM iodoacetamide, 2 mM phenylmethylsulfonyl fluoride, and complete mini protease inhibitor cocktail (Roche Applied Science, Sigma-Aldrich), by incubating the cells at 4 °C for 30 minutes. Total protein concentration was determined using the BCA Protein Assay (Pierce, Thermo Fisher Scientific). Between 10 and 30 μg of total protein was loaded onto 8%, 10%, or 12% SDS-PAGE gels under reducing conditions and transferred to nitrocellulose membranes (Amersham, Sigma-Aldrich). Membranes were blocked in PBS containing 0.1% Tween-20 and 5% fat-free milk and incubated overnight at 4 °C with primary antibodies at 0.5 μg/ml in the same blocking buffer but with 2.5% fat-free milk. Horseradish peroxidase–conjugated F(ab’)_2_ fragment goat antimouse IgG or rabbit antigoat IgG secondary antibodies (Jackson ImmunoResearch Laboratories) were used at 1:5000 for 1 hour at 4 °C. Primary antibodies included anti-ACE2, clone AC384 (AdipoGen Life Science/Coger, Paris, France); anti-TMPRSS2, clone S20014A; anti-NRP1, clone 14H4; anti-CD147, clone HIM6; anti–keratin 10, rabbit polyclonal Poly19054 (BioLegend); anti-CTSL, clone 33/1 (eBioscience, Thermo Fisher Scientific); anti-AXL, polyclonal goat IgG AF154; and anti-DPP4, polyclonal goat IgG AF1180 (R&D Systems, Bio-Techne SAS, Noyal Châtillon, France). Antibody binding was detected using SuperSignal West Femto (Pierce, Thermo Fisher Scientific) and visualized on a Fusion-FX Imager (Vilber). Membranes were stripped in Restore Plus Western Blot Stripping Buffer (Thermo Fisher Scientific) according to the manufacturer’s instructions and reprobed with anti–β-actin polyclonal rabbit antibody (Sigma-Aldrich) at 0.2 μg/ml, followed by horseradish peroxidase–conjugated F(ab')2 fragment rabbit antigoat IgG secondary antibody (Jackson ImmunoResearch Laboratories) at 1:10,000. Band intensities were quantified using ImageJ (version 1.53t) prior to saturation and normalized to β-actin.

### Flow cytometry

Adherent cells were detached using Accutase solution (1X) (Sigma-Aldrich). Fc receptors were blocked with 10% normal mouse serum and 100 μg/ml human Ig (Sigma-Aldrich) in staining buffer (PBS containing 0.5 mM EDTA, 0.1% BSA, and 0.05% sodium azide) for 30 minutes at 4 °C. Cells were then incubated with fluorescently labeled antibodies against ACE2 (clone Poly5036), TMPRSS2 (clone S20014A), and DPP4 (clone BA5b) (BioLegend); NRP1 (clone U21-1283), CD147 (clone HIM6), and AXL (clone 108724) (BD Biosciences, Le Pont de Claix, France). Staining with unlabeled anti-ACE2 antibodies (clone AC18F, AdipoGen Life Science/Coger; clones 171607, 379131, and 535919, R&D Systems; and clone Poly5036, BioLegend) was followed by incubation with fluorescently labeled donkey antimouse or antigoat IgG secondary antibody (Invitrogen, Thermo Fisher Scientific). Staining with biotin-conjugated recombinant SARS-CoV-2 Spike Protein S1 (RBD) (AdipoGen Life Science/Coger) was followed by incubation with fluorescently labeled streptavidin (BD Biosciences). Blocking anti-ACE2 mAb (clone AC384) or mIgG1 isotype control were added 30 minutes prior staining with SARS-CoV-2 Spike Protein S1 (RBD). Samples were acquired on a BD LSR Fortessa flow cytometer (BD Biosciences) and analyzed using DIVA (version 8) and FlowJo (version 10) software (BD Biosciences).

### IF

A549, A549-hACE2, Calu-3, Caco-2/TC-7, N/TERT-2G, NHDF, and NHEK cells were seeded on glass coverslips at a density of 5 × 10^5^ cells per well in a 12-well plate and allowed to adhere for at least 24 hours. Cells were fixed in cold 4% paraformaldehyde for 10 minutes, washed 3 times with PBS, and blocked for 1 hour in PBS containing 3% BSA and 10% normal donkey serum. Fixed cells were stained with a primary antibody against ACE2 (clone Poly5036) (BioLegend) at 1:200 dilution for 1 hour and 30 minutes, followed by Alexa Fluor 555-conjugated donkey antigoat IgG secondary antibody (Invitrogen, Thermo Fisher Scientific) at 1:500 dilution and DAPI at 1:2500 dilution. For spike binding, cells were blocked using the endogenous avidin/biotin-blocking kit, according to manufacturer’s instructions (Invitrogen, Thermo Fisher Scientific). Soluble recombinant SARS-CoV-2 Spike Protein S1 (RBD) (rec.) (His) (Biotin) (Adipogen Life Science/Coger) was incubated at 1:100 dilution for 1 hour and 30 minutes at room temperature and washed 3 times with PBS. Cells were fixed in cold 4% paraformaldehyde for 10 minutes, washed in PBS, and stained with Alexa Fluor 555-conjugated Streptavidin (Invitrogen, Thermo Fisher Scientific) at 1:500 dilution and DAPI at 1:2500 dilution. Cells were mounted using Fluoromount Gold (Invitrogen, Thermo Fisher Scientific). Fluorescent images were captured on a Zeiss Axioplan 2 piloted by Micro-Manager (United States National Institutes of Health). All images were deconvolved with Huygens Remote Manager (version 3.8) (Scientific Volume Imaging).

### SARS-CoV-2 virus, infection, RT-qPCR, and IF

The Delta strain (B.1.617.2) hCoV-19/France/HDF-IPP11602i/2021 was provided by the National Reference Centre for Respiratory Viruses, hosted by Institut Pasteur (Paris, France) and led by Sylvie van der Werf. The human sample from which the strain was isolated was obtained from Raphaël Guiheneuf (CH Simone Veil, Beauvais, France). This strain and a Wuhan variant were also supplied through the European Virus Archive goes Global platform, a project funded by the European Union's Horizon 2020 research and innovation program under grant agreement number 653316. The virus was propagated in VeroE6 cells cultured at 37 °C with 5% carbon dioxide, in DMEM supplemented with 25 mM 2-[4-(2-hydroxyethyl)piperazin-1-yl]ethanesulfonic acid, and harvested 72 hours after inoculation. Virus stocks were stored at −80 °C. Viral titers were determined using plaque assays (Laplantine et al, 2022; Lyonnais et al, 2021) and expressed as plaque-forming units per milliliter. All work with infectious SARS-CoV-2 was conducted in a certified BSL3 (biosafety level 3) facility by trained personnel at CEMIPAI UAR3725. For antiviral drug and antibody treatments, cells were pretreated for 2 hours before SARS-CoV-2 infection with either anti-ACE2 blocking antibody (clone AC384, AdipoGen Life Science/Coger), remdesivir (10 μM) (Tebubio SAS, Le Perray en Yvelines, France), camostat (20 μM), E64D (20 μM), or DMSO as vehicle control (Sigma-Aldrich). Infections were performed at MOI of 0.01 for the control A549-hACE2 cell line; MOI of 1 for A549; and MOI of 0.01, 0.1, and 1 for NHEK and N/TERT-2G for 2 hours at 37 °C. The virus inoculum was removed, and cells were washed in PBS and incubated in culture medium for 24, 48, and 72 hours before collection of cell supernatant and lysis of cells. Infection with SARS-CoV-2 strain hCoV-19/France/OCC-IHAP-VIR13/2021 was performed by NeoVirTech (Toulouse, France). Briefly, VERO E6 and NHEKs were infected at an MOI of 0.5 with SARS-CoV-2 strain hCoV-19/France/OCC-IHAP-VIR13/2021 for 2 hours, and the cells were washed 3 times and incubated in culture medium for 72 hours. Viral titers in the collected supernatants were determined by standard cytopathic effect assay on VERO E6 using Spearman & Käber algorithm and expressed as 50% Tissue Culture Infectious Dose (TCID50)/ml (NeoVirTech).

#### RT-qPCR

Total cellular RNA from infected (denoted as INF) and noninfected (denoted as NI) cells were collected in RLT buffer and extracted using the RNeasy Mini Kit (Qiagen, Redwood city, CA), following the manufacturer’s instructions. Viral RNA was quantified in triplicate by qRT-PCR using primers targeting the E gene of SARS-CoV-2 (E_Sarbeco-F ACAGGTACGTTAATAGTTAATAGCGT; E_Sarbeco-R-ATATTGCAGCAGTACGCACACA) (Sigma-Aldrich) (Corman et al, 2020; Laplantine et al, 2022; Lyonnais et al, 2021). Reactions were performed using the Luna Universal One-Step RT-qPCR Kit (New England Biolabs, Evry, France) on a LightCycler 480/1536 (Roche). Calibration was carried out using the nCoV-E-Sarbeco-Control Plasmid (Eurofins Genomics). Gene expression of GAPDH was quantified in triplicate using the same Luna kit and PCR assay (GAPDH forward GCTCACTGGCATGGCCTTCCGTG; GAPDH reverse TGGAGGAGTGGGTGTCGCTGTTG). Relative gene expression was calculated either by normalization to GAPDH levels or to total RNA input (20–40 ng), as indicated in the figure legends. Quantification was expressed as copy number of gene E (copy/μl) or through the ΔCt method, with results presented as log_2_ fold change relative to the gene median. Absolute quantification of the viral E gene was performed in triplicate using the RT-qPCR protocol described earlier, with results compared with a standard curve. Data are presented as viral RNA concentration in copy/μl.

#### IF

Cells were seeded on glass coverslips and infected with the SARS-CoV-2 Wuhan strain at an MOI of 0.01 for A549-hACE2 cells or an MOI of 1 for NHEK cells. Seventy-two hours after infection, cells were washed with PBS and fixed in 4% paraformaldehyde in PBS for 15 minutes at room temperature, followed by permeabilization with 0.2% Triton X-100 in PBS for 4–5 minutes and blocking in 2% BSA in PBS for 15 minutes. Cells were then incubated with rabbit anti–SARS-CoV2 membrane (M) protein antibody (clone 100-401-455, 1:250) (Rockland, Tebubio SAS) for 2 hours at room temperature. After washing with PBS, cells were incubated with Alexa Fluor 568–labeled goat-anti-rabbit secondary antibody (1:500) (Invitrogen, Thermo Fisher Scientific) for 2 hours at room temperature. Mounting media prolong gold antifade reagent with DAPI (1:5000) (for nucleus staining) was used for confocal microscopy (Thermo Fisher Scientific). Images were acquired using a Zeiss Cell Discoverer 7 laser-scanning confocal microscope equipped with a ×20 objective (Carl Zeiss Microscopy GmbH, Oberkochen, Germany).

### Statistical analysis

Statistical analyses were performed using GraphPad Prism 10 software (GraphPad Prism, San Diego, CA). Statistical significance versus 1 was evaluated using the Wilcoxon signed-rank test. Other comparisons were performed using the Kruskal–Wallis 1-way ANOVA test. A *P* < .05 was considered statistically significant.

## Ethics Statement

Human skin samples, normal human epidermal keratinocytes, reconstructed human epidermis, and normal human dermal fibroblasts were purchased from Sterlab (Vallauris, France). Skin samples were obtained from abdominal plastic resection surgery, with the patient’s written informed consent, in accordance with the Declaration of Helsinki (Sterlab AC-2023-5620), and under French Ethics Committee approvals (DC-2023-5457 and DC-2018-3312).

## Data Availability Statement

All data supporting the conclusions of the manuscript are shown in the corresponding figures and supplements. No large datasets were generated or analyzed.

## ORCIDs

Leslie Hertereau: http://orcid.org/0009-0003-0731-5750

Manon Barthe: https://orcid.org/0000-0002-0657-9194

Noura Lamghari: http://orcid.org/0009-0002-4655-9945

Peggy Merida: http://orcid.org/0009-0001-5613-2745

Gaelle Pommier: http://orcid.org/0009-0003-1557-7910

Elisabeth Pinel: http://orcid.org/0009-0004-7695-609X

Jitendriya Swain: http://orcid.org/0000-0003-2921-0688

Delphine Muriaux: http://orcid.org/0000-0001-8517-9342

Hanan Osman-Ponchet: http://orcid.org/0000-0003-4994-5673

Veronique M. Braud: http://orcid.org/0000-0001-8213-3947

## Conflict of Interest

The authors state no conflict of interest.

## Figures and Tables

**Supplementary Figure 2:**